# Why Do We Need Computational Models of Psychological Change and Recovery, and How Should They Be Designed and Tested?

**DOI:** 10.3389/fpsyt.2020.00624

**Published:** 2020-06-30

**Authors:** Warren Mansell, Vyv Huddy

**Affiliations:** ^1^ School of Health Sciences, University of Manchester, Manchester, United Kingdom; ^2^ Clinical Psychology Unit, University of Sheffield, Sheffield, United Kingdom

**Keywords:** functional model, psychotherapy, mechanism of change, Perceptual Control Theory (PCT), dynamic models

## Abstract

Traditional research methodologies typically assume that humans operate on the basis of an “open loop” stimulus-process-response rather than the “closed loop” control of internal state. They also average behavioral data across repeated measures rather than assess it continuously, and they draw inferences about the working of an individual from statistical group effects. As such, we propose that they are limited in their capacity to accurately identify and test for the mechanisms of change within psychological therapies. As a solution, we explain the advantages of using a closed loop functional architecture, based on an extended homeostatic model of the brain, to construct working computational models of individual clients that can be tested against real-world data. Specifically, we describe tests of a perceptual control theory (PCT) account of psychological change that combines the components of negative feedback control, hierarchies, conflict, reorganization, and awareness into a working model of psychological function, and dysfunction. In brief, psychopathology is proposed to be the loss of control experienced due to chronic, unresolved conflict between important personal goals. The mechanism of change across disorders and different psychological therapies is proposed to be the capacity for the therapist to help the client shift and sustain their awareness on the higher level goals that are driving goal conflict, for sufficiently long enough to permit a trial-and-error learning process, known as reorganization, to “stumble” upon a solution that regains control. We report on data from studies that have modeled these components both separately and in combination, and we describe the parallels with human data, such as the pattern of early gains and sudden gains within psychological therapy. We conclude with a description of our current research program that involves the following stages: (1) construct a model of the conflicting goals that are held by people with specific phobias; (2) optimize a model for each individual using their dynamic movement data from a virtual reality exposure task (VRET); (3) construct and optimize a learning parameter (reorganization) within each model using a subsequent VRET; (3) validate the model of each individual against a third VRET. The application of this methodology to robotics, attachment dynamics in childhood, and neuroimaging is discussed.

## Introduction

It is well recognized that randomized controlled trials, in isolation, cannot identify the mechanism of action of a psychological therapy (1–3). Most commonly, tests of mechanism involve prospective studies or experimental designs (3, 4). In an earlier review, we identified a number of fundamental limitations with using these methods in psychology, despite their almost universal acceptance (5). In the current article, we will begin by explaining how these limitations specifically apply to research on the mechanisms of psychological therapy. We then propose an alternative method that addresses these issues—building and testing computational models against real-world data. Arguably, this is the ultimate test of a theory—that it specifies a working model that has identical properties to the real system. We will share a number of methodologies and initial findings of this work, utilizing an integrative psychological framework known as PCT (6–9).

## What Are the Limitations of Existing Methodologies Within Psychological Therapies?

A case has been made regarding the limitations of randomized controlled trials of therapies in being able to understand how a given psychological intervention has its effects (1–4). Psychological interventions are typically complex, multifaceted treatments, and so in a typical controlled trial, it is not clear which of the various active components of the treatment could account for any differences. The situation can be improved by tweaking a certain element of the intervention and comparing the different versions of the intervention experimentally (3, 4). However, there is little evidence that this approach has yielded insights into more effective treatments (10). The element of trial and error in this process means progress is likely to be slow and expensive if a significant proportion of experimental trials do not yield improvements.

While this research provides a window on potential mechanisms of change, these studies are based on a number of assumptions regarding the nature of human behavior and scientific research that challenge the interpretation of their findings (5, 11, 12). These issues can be summarized in four points. First, there is a lack of appreciation within current research design that behavior continuously feeds back on sensory input (such as when you move your head and eyes to continuously change what you are perceiving within your surroundings). Second, there is a lack of theories that acknowledge the continuous and dynamic variation in behavior at the level of the individual, leading to methods that artificially choose to “chunk” or average measurements of behavior instead. Third, there is a disjunct between making an inference about an internal mechanisms of change in psychological therapies within an individual, and the use of group statistics to research the efficacy of psychological therapies on the “average” of a group of individuals, or to assesses statistical trends in a variable (e.g., individual differences in a putative mechanism of change) across a group of individuals. Mediation analyses attempt to determine what variables are pertinent to change. For example, mindfulness may have an effect on outcome *via* rumination and worry (13). However, this approach examines group statistics to understand individuals. An established line of research on the relationship between self-efficacy on performance has found this can lead to opposite relationships at the level of group averages and individuals. Individual differences in self-efficacy demonstrate a positive association with performance compared to analyzing change in self-efficacy within individuals—where the opposite relationship is shown to occur (14). Thus, studies of this kind show that it is possible for group statistics to generate erroneous conclusions regarding the processes occurring within the individual.

Fourth, most theories of psychopathology and the mechanisms of psychological therapies are described verbally. Even if they are operationalized through a diagram, this is rarely in a form that could form a working model that would be necessary to test it against real-world data (15).

The traditional approach to psychological therapy research contrasts with progress of the physical sciences and engineering, and to some extent biology. They have utilized functional model building (16). For example, the theory of aerodynamics is used within computer simulations to model the flight ability of a new aircraft prior to manufacturing. The accuracy of these models is extremely high, which is required in order to assure their feasibility, safety and economic performance. With the fast pace of development of both virtual reality and robotic systems, there is now the potential for models of behavior and cognition to be tested with similar precision (5).

## How Can Computational Modeling Provide Robust Tests of Psychological Theories of Therapy?

The computational modeling approach requires the mathematical specification of a theory. The challenge this poses is how a theory of psychological change can be described in mathematical terms. In a key article on this topic, Moutoussis and colleagues (17) argue that since psychological therapy requires learning, this allows the computational understanding of learning to be applied to psychological change in therapy. They go on to highlight how learning and inference is central to how Cognitive Behavioral Therapy (CBT) has been conceived from its development onward. One example that Moutousssis and colleagues expand on in their paper is the mapping of inferential distortions to problematic beliefs (17). They provide an example of how a computational model of avoidance learning can be applied to understanding exposure and response prevention. This model specifies key inferences about the outcomes of behavior as the main variables in the models and can be compared to actual behavior. We will return to the modeling of learning later in the article.

There is also a body of earlier work that has focused on the modeling of appraisal and affect that could be applied to psychotherapeutic change (18, 19). Some of these appraisal models have extended their reach to artificially intelligent systems and complex robotic devices (20). There are also computational models of individuals that model the dynamic relationship between constructs used in cognitive case conceptualizations such as catastrophic thoughts, avoidance, arousal, and cognitive restructuring (21, 22).

Returning to the suitability of computational modeling of beliefs, there is indeed considerable evidence that negative, catastrophic or self-critical beliefs are associated with self-reported distress (23). However, there are also reasons to suggest that personal goals (values, principles, standards, and ideals) may be more fundamental. For example, there is evidence that the content of intrusive experiences such as memories (24) and auditory hallucinations (25) are closely associated with personally valued goals, and that the degree of distress associated with auditory hallucinations (26), and phobic avoidance (27) is associated with how much they interfere with people’s more important, self-definitional goals. As will be described below, another way of conceiving psychological change is to consider it as loss and restoration of control. We will introduce and explain perceptual control theory (PCT) because it provides a definition of control that specifies biologically feasible, mathematical models of control that can be applied to the complexities of the mechanism of psychological therapy.

PCT is derived from control engineering (28), which in turn had its basis within the homeostatic systems of the body (29). According to PCT, control is the achievement and maintenance of a variable at a preselected state through actions that counteract disturbances to that variable (9). This is carried out through circular feedback process between the individual and their environment. The current perception of an aspect of the self or environment (e.g., noise level) is compared to the *reference value* for this variable (e.g., a quiet level). The discrepancy between the two (the *error*), is amplified (by a *gain* factor) and drives actions (e.g., to leave the room) that counteract disturbances (e.g., other people talking loudly) to try to keep the variable at its desired level. PCT proposes that we hold our reference values for a whole range of perceptions of ourselves and the world, and that all our actions are aimed at keeping these close to their preferred level—their “just right” states. People are not necessarily conscious of this process; indeed Powers (9) proposed that people are only aware of a small number of perceptual variables that they are controlling at any one time. It is also important to note that reference values are not necessarily fixed, but can be altered internally as necessary when circumstances change; we will discuss this mechanism when we introduce hierarchies later on. Sometimes, people are unable to keep perceived aspects of the environment in a desired state because they do not have the resources (known as the feedback function in PCT) to do so. These kinds of changes in the environment are termed insuperable disturbances. These factors would be beyond the individual’s control and experienced as distressing. For example, it would not be possible to bring back to life a deceased family member, or for a neglected infant to find more capable caregivers.

**Figure 1:**
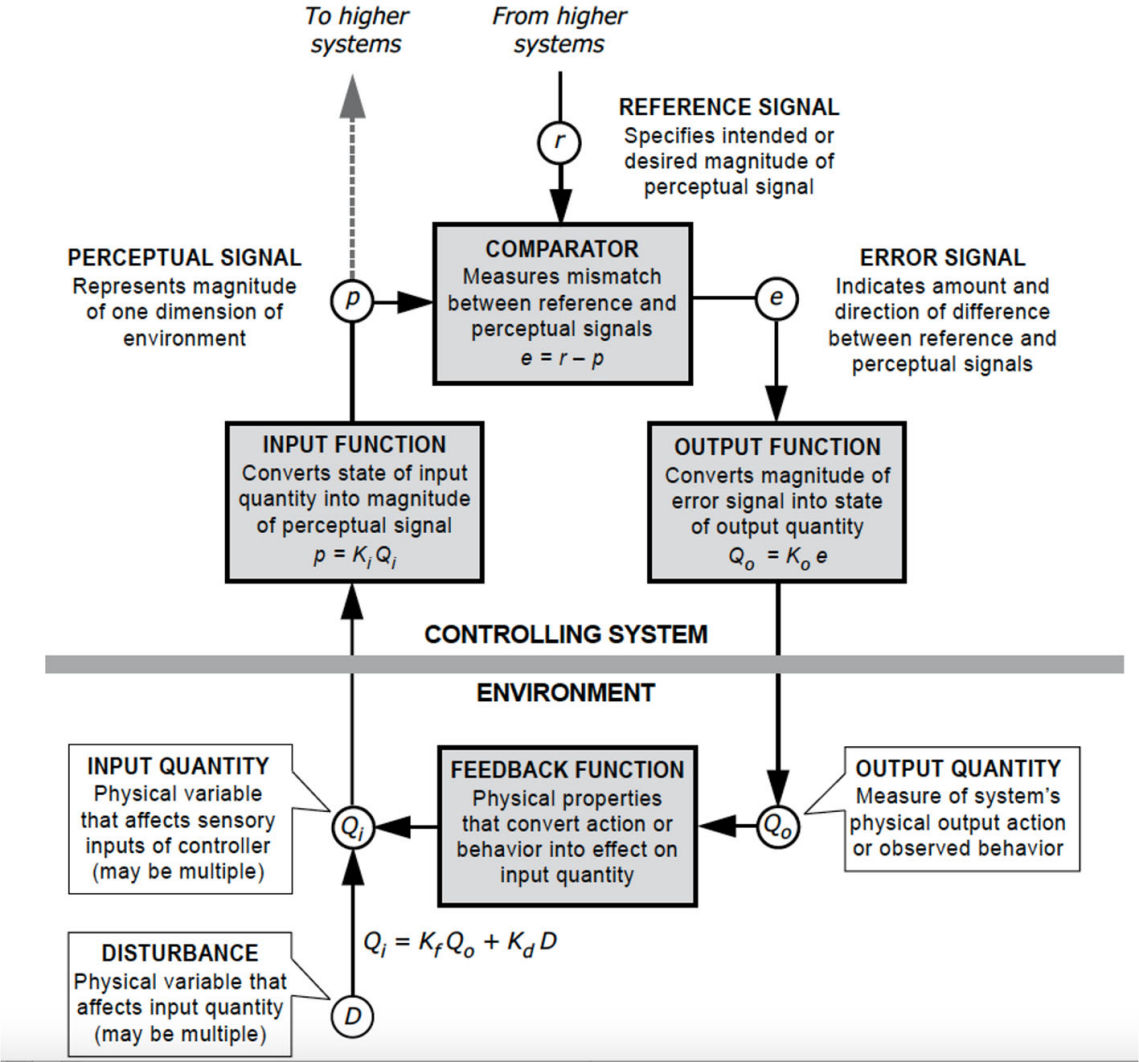
Diagram of the control unit according to perceptual control theory. Permission granted from Dag Forssell, redrawn from an original figure by William T. Powers.

**Figure 1** shows in more detail the components of a single control unit. This is the basic building block of the theory. The diagram clarifies a number of features. Importantly, it shows the boundary between the organism and the environment, with a clear indication that the reference value (r) is situated inside the individual. It is set by the output of another control unit that is situated on the level above; in other words the individual can set and modify their own goals. Within a control unit, an input function (Ki) transforms a physical variable in the environment (Qi) into a perceptual input (p). It is this perceptual signal that is controlled. First, it is subtracted from the reference value to generate an error (e). This is amplified by an output function (Ko) to transform it into an output quantity (Qo) that acts in the environment. It acts *via* the feedback function in the environment (Kf) that counteracts the disturbances in the environment (D) to affect the input quantity such that p is kept as close to r as possible.

PCT utilizes the Test for the Controlled Variable [TCV; (9)]. Mansell and Huddy (5) summarize this approach as follows:

“…the experimenter aims to set up a design in which the perceptual variables that the participant controls can be inferred by the effect of their actions the controlled variable which is observed by the experimenter … the experimenter needs to (a) characterize the continuous array of perceptual information that is available to the participant through their senses (b) identify or provide the means through which the participant has to control their perceptual input within the environment, and (c) identify or manipulate the disturbances to this control.” (p. 310, 5).

These models have been applied across wide domains of psychology. For example, they have been designed to closely emulate tracking a cursor on a computer screen (30), movement across a baseball pitch to catch a flyball (31), and selection of work schedules for nurses (32). In each case, this evidence is consistent with a PCT explanation of human behavior. An important contemporary advance on these models is that they can be used to reliably distinguish between individuals; a “control profile” of each individual can be extracted, at least within the domain of simple motor control (33). However, regardless of whether these conclusions can be critiqued, there remains the question of how models of this kind can be applied to the complexities of change through psychological therapies.

## The Core Principles of PCT Involved in Psychological Distress and Psychological Change

A series of articles have begun to make the case that a PCT explanation of psychological change is supported by existing computational models (2, 34, 35). These accounts rely on three additional principles within PCT that help to model how psychological change occurs in therapy. These are *hierarchies*, *conflict*, and *reorganization*.

The existence of hierarchies is possibly the simplest to explain, because the concept of a hierarchy within the brain and behavior is common across psychology and neuroscience (36). It is also core to most theories of goals in psychology (37). Hierarchies of control provide a way to organize the many variables that a person strives to control in their life. It also allows flexibility in terms of providing multiple means (lower levels goals) to an end (higher level goal). Thus, Powers et al. (7, 8) proposed that control systems are organized in a cascading, branching hierarchy with the more abstract, fundamental variables (e.g., self-worth and honesty) toward the top, and the more concrete, malleable variables toward the bottom (e.g., perception of proximity to another person, perception of motion). By the 1990s, he had specified eleven levels of this hierarchy through detailed introspection, and each level potentially corresponds to specific fields of psychological inquiry (e.g., sequencing, if-then programming, abstraction of principles). The hierarchy is an essential component to understand how the principles of conflict and reorganization are applied to understand, and model, psychological change.

According to PCT, the psychological distress that entails that a person seeks therapy is a manifestation of loss of control (6–8). It is not, for example, the existence or conviction level of a specific belief, or nature of the inferences that a person makes. The loss of control maybe acute and extreme as in a panic attack, insidious, and biological as in anorexia nervosa, morally threatening such as in obsessions and compulsions, or profound and existential such as in a psychotic breakdown. There are a number of fundamental variables that any human needs to control, and distress is experienced when these are in *error*—when they deviate from their *goal state* or *reference value*. For example, chronic physiological arousal would deviate from the desired state of calmness. An important subset of these fundamental variables are (unlearned) *intrinsic variables*—those maintained by biological control processes in the brain and body—such as body temperature, blood glucose, sleep-activity states, and freedom from pain. Some of these variables will be shared in common with other people, but each individual will inevitably have a different range of controlled variables with differing priorities. The loss of control maybe acute and extreme as in a panic attack, insidious, and biological as in anorexia nervosa, morally threatening such as in obsessions and compulsions, or profound and existential such as in a psychotic breakdown. Indeed, it may possible to map different symptoms of mental health disorders onto specific levels of the perceptual hierarchy. For example, within depression, worthlessness would be experienced at a highest level—the system concept; guilt would be experienced at the principle level; and inability to plan and engage in everyday routines would be experienced at the program level.

People will also differ in terms of the degree of interference, or *conflict*, between the variables they strive to control. For example, in a study of hearing voices in clinical and non-clinical participants, it was the interference between voice hearing and an individual’s personal and uniquely specified goals that corresponded with their distress about voice hearing, over and above various properties of the voices (26). Indeed the diagnostic label given to a person’s mental health problem may be in a large part explained by the personally important realm of their life in which they have lost control (12).

Following the above account, there are two ways in which psychological distress can be alleviated: by directly enhancing control, and by resolving conflict (35). One successful example of a way to enhance control is to provide patients with the opportunity to determine the timing, appointment scheduling, session length, and duration of their psychological therapy (38). Further successful examples include providing people with the control of the topic of each therapy session (39), or providing them with technology to exert moment-by-moment control of their level of exposure during therapy (40).

Despite the success of addressing control problems directly, helping patients in this way will not be sufficient to address problems of conflict. According to PCT, the majority of cases of chronic loss of control, and therefore long-lasting psychological distress, can be traced to issues of conflict, whether between, or within, individuals (9, 34). Conflict occurs when people attempt to control two or more opposing standards for the same variable. In the case of a specific phobia, for example, the patient may both want to get closer to what they are afraid of in order to overcome their fears and be a strong person, but at the same time want to get further away in order to stop a catastrophic event such as being harmed or humiliated (6). Yet, the experience that the person is trying to control can be completely internal, such as the vividness of memories. On the one hand, a traumatized individual may want to forget their trauma to try to remain sane and get on with their life, but on the other hand they may want to remember their trauma in detail to be a credible witness in court and get justice.

It is notable that Powers’ (9) theory has been used to generate a model of obsessive compulsive disorder (41). This model also focused to the role of goal conflict in maintaining distress, but it did not describe how the conflict would be resolved within Powers’ (9) theory. It also suggested that there could be a “faulty comparator” deficit at the heart of the condition. There are a number of sophisticated neural network and robot models that test deficit models of psychiatric disorders, and that do not directly examine the process of psychological change that would occur within therapy [e.g., (42, 43)]. In particular, given the heritage of Lewis et al.’s (42) robot within Pitman’s (41) use of PCT, it provides a good embodied grounding for future research that might model the resolution of conflict through reorganizations.

The concept of conflict, and conflict resolution *via* insight, has its origins at least as far back as the early psychoanalytic theories (44, 45). Indeed, computational psychoanalysis has formed its own recognizable discipline (46). However, unlike PCT, the original psychodynamic theory does not provide the formal structure required for computational modeling, and later formalisms are used for computational modeling. Yet, in contrast to psychoanalysis, conflict has had an operational definition within PCT since its inception (7, 8), as the specification of opposing reference values for the same perceptual variable; PCT also provides a specified hierarchical architecture, the mathematical specification of its components, and the learning algorithms to model how conflict is resolved, as we elaborate below. It is these kinds of conflicts that are target by a therapy based on PCT, known as Method of Levels (47). Yet, according to PCT, all effective psychological therapies work through resolving conflict, but to varying levels of efficiency (12).

**Figure 2:**
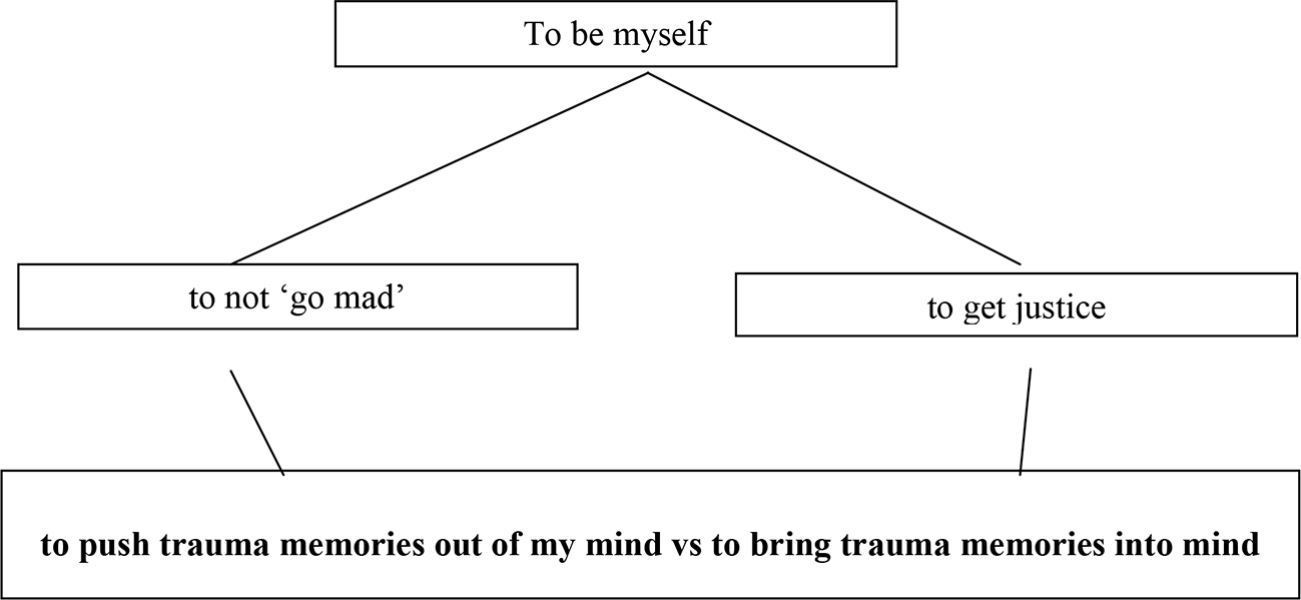
A diagram to illustrate the three levels involved in the loss of control due to unresolved conflict according to PCT. The top level contains a single higher level system that sets two or more subgoals at the middle level. These subgoals send out conflicting references for a low level system that entails loss of control and instability—in this case, the recall and suppression of trauma memories. Note that this model is simplified—there will be control systems intermediate between the “mid” and “low” level, such as to “to be able to describe my trauma” and “to be a credible witness”, as a means to achieve justice through the recall of trauma memories.

Conflict cannot be solved merely by helping a person to control. The above clinical examples illustrate that actually, both sides of a conflict may be important and worthwhile to the individual—to be a strong person and to be safe; to stay sane and to get justice (see **Figure 2**). The conflict cannot be solved by helping to promote one side. Something novel and innovative needs to occur. This is where reorganization comes in. Powers and colleagues (7) adapted an idea from an early cybernetics pioneer (48) and applied it to the PCT model. When loss of control is experienced for any length of time, it engages a system that creates trial-and-error changes to the properties of the control system in error. In order to resolve conflict, these changes need to be directed not at the two goals in conflict, but above them, to the higher-level system that is setting the reference values for the two goals. So, for example, the client who is in two minds about whether to face their fears or continue to avoid them, may ultimately, be trying to be a good husband and father by staying safe and avoiding threat, and by being strong and facing fears over time.

Reorganization is also the fundamental learning algorithm in PCT. Outside PCT, learning is typically modeled as associative learning or reinforcement learning. Within associative learning, stimuli in the environment are associated with one another when they have a temporal or contingent relationship with one another. Within reinforcement learning, specific behaviors are reinforced when they lead to increased reward or reduced punishment or aversive experience in a pattern known as the reinforcement schedule (49). There are highly reliable relationships between responses and the reinforcement schedules being used in a given experiment (50). From a PCT perspective, this is to be expected because the organism is attempting to maintain the intake of sustenance at the preferred reference level. Crucial to this from a PCT perspective is that it is not the action that is reinforced but the perceptual effects of action that the organism seeks to maintain. Marken (51) used an experimental set up where bar pressed away from the goal were equally likely to those toward the goal (i.e., all responses were equally reinforced). Crucially, responses became more likely as goal progress deteriorated, regardless of the specific consequences of each action. Similarly, research on body movements shows that preferred distance can be achieved by flexion or extension regardless of the specific muscle movements involved (52). Actions must vary rapidly when changes in the environment occur which alter the effects actions have on the environment [e.g., using a reversal learning task; (53)]. When such changes in the reinforcement schedule occur, behavior has to vary to ensure the preferred intake of sustenance is maintained. In these circumstances, PCT utilizes a learning algorithm known as reorganization. It works by generating random changes in the parameters of control systems when error is sustained, until the error is reduced to a near zero level (see later for more detail). Behavior changes are observed during reorganization but no specific stimulus-behavior associations are reinforced. Nonetheless, the results of reorganization modeling lead to the observations that most other researchers would describe as instances of reinforcement learning (54, 55).

PCT proposes a specific pathway to the resolution of conflict in practice, mediated by the focus of awareness. A client is given the opportunity to talk about a problem. It is assumed that what a client talks about is what is currently the focus of awareness, and that significant, enduring error attracts awareness. Next, it is proposed that while focusing on this problem, awareness shifts to the “mid-level” goals in conflict that are responsible for this lower level problem. The therapist can aid this process through asking about disruptions indicating transient shifts in awareness as the person is talking. Finally, and importantly, it is proposed that awareness shifts and sustains on the high-level goal (or control system) that is setting the two incompatible mid-level goals. Once here, the therapist’s job is to help the client stay at this higher level while reorganization has its effects. Because reorganization operates through trial-and-error, it takes an unspecified amount of time, and it may make changes that do not improve control, until it ultimately reaches a higher level organization that resolves the conflict and improves control, thereby reducing distress. **Figure 2** shows an example of how goal conflict can be represented at three levels.

The above account is detailed, verbally described, and somewhat linear in nature, going against some of the recommendations we had in mind for the future of psychotherapy research. Indeed, we have published studies that support the above account, but they are based on subjective, observer coding of the current focus of awareness in patients receiving Method of Levels (56, 57). So, the question remains, could such a sophisticated process, based on these four principles, be testable through computational modeling? We will first describe how these principles have been tested in isolation, and then how they have been applied together to more directly emulate psychological change.

## Overview of the Methodology and Implementation of PCT Computational Models

The methodology and implementation of the computational models informed by PCT can be classified in various ways as one moves from principle to practice. These features are described and reviewed in detail for models of manual tracking in a recent systematic review (58).

First, there is the question of whether the validity of the model is judged by (a) its qualitative similarity to real-world data, (b) its quantitative fit with real-world data and (c) its superior fit to real-world data compared to a model informed by a competing theory. In the sections to follow, we introduce some examples of (a), many examples of (b), but very few studies have tested (c) comparative validity.

Second, there is the question of which elements of PCT are incorporated into the model. In most instances, the models involve all of the functions, signals and quantities shown in **Figure 1**. Some are often more detailed however; for example, the delays of signals as they permeate around the loop may be incorporated, and the *output function* may involve a gain (amplification), a slowing function (such as a “leaky integrator”) and other transformations to create the current output quantity. Many computational models also involve further elaboration going beyond the simple control unit—hierarchies, conflict, and reorganization are not shown explicitly in **Figure 1** and yet they form key components of the theory (9).

Third, there is the question of which software is used to carry out the modeling. The platforms vary widely and so we reference here some key examples for further reading. First, some of the earliest models were essentially algebraic equations [e.g., (30)]. Later, models used the “function” property of excel spreadsheets to make iterative computations (59). Some groups of researchers have used visual model-making platforms such as Vensim (32), or they have created visualizable models through programming code, such as C++ (54) or Matlab (33, 58). Finally, there bespoke platforms that have the workings of a control unit pre-specified, and the user creates a connected network of these units and specifies the parameters. An example of a bespoke PCT platform will be introduced later in this article.

## Separate Computational Models of Hierarchies, Conflict, and Reorganization

An earlier article summarized a number of computational models of each of the principles we have described (2). First, there are a number of PCT models that utilize a hierarchical organization to model the control of physical variables. An early study utilized a two-level hierarchy to model how human participants controlled the relative distance of two lines on the screen using two control handles (60). These movement of the cursors utilized by these models was highly correlated with the movements made by human participants, at a level close to r = .99. A hierarchical model involving four levels has been used to simulate the balancing of an inverted pendulum (61). Both of these studies show the utility of hierarchical control, but they do not show that it is necessarily superior to control without a hierarchy in modeling human behavior. Within our research group, we have produced initial evidence that this may be the case. We designed a model of visual manual tracking that required two levels—the control of proximity of a cursor to a target was achieved by setting reference values for the velocity of the cursor (62). We then optimized a different model to each of 24 participants’ data, and compared the fit to a single layer, position control model. The hierarchical model was superior in fit to a non-hierarchical proximity control model when the target moved in a predictable (sinusoid) pattern, but similar in fit with a less predictable (pseudorandom) pattern. Most recently, we have confirmed that the PCT hierarchical model of the inverted pendulum within a robotic device shows superior balance in the presence of disturbances compared to two other popularly used (non-hierarchical) controllers for the same robot (63).

A number of authors have modeled conflict to illustrate its properties, and point to parallels in everyday human behavior [e.g., (34, 54, 64, 65)]. For example, Carey (34) used models of individual PCT agents initially designed to illustrate crowd behavior [e.g., (66)]. One of these agents, A, had a goal to move to a fixed location but to stay away from another agent, B. B had a goal to get close to A. Carey (34) explored the effects of a parameter known as *gain*, which is effectively the effort put in to achieve one’s goals. At low levels of gain in both agents, there was no evidence of conflict, with both agents reaching their goals. However, when the gain of B was increased, A could not reach its goal because it was blocked by B. This could illustrate the suppression of one goal (e.g., to never feel angry) by another (e.g., to express one’s anger). If, in turn, the gain of A was increased, the two agents oscillated widely and vigorously, potentially illustrating the loss of control that can come from increasing one’s efforts. The remaining studies of conflict have not interpreted their findings in the context of psychological distress, but either in terms of social group behavior (63, 64), or to illustrate more universal principles (54).

An early model of reorganization illustrated that it followed a similar principle to that used by human participants to reach a goal when only the timing of a trial-and-error change in behavior could be controlled (66). Probably, the most sophisticated computational models of reorganization published to date illustrated how it reduces conflict and improves control within a simulated model of an arm with 14 independent control systems governing the movement at joints (54). At the start of the simulation, the control systems interfered with one another. However, after a period of reorganization in which the gain of each system is reset randomly when error across the whole arm increases, they each converge to a gain that counteracts any disturbances from other joints, and the whole arm is able to smoothly execute its movement—one that Powers selected from Tai Chi.

Powers (54) clearly illustrates the capacity for reorganization to reduce error, but no attempts were made to quantitatively test this model against real-world behavior. There are some key parallels between observations of the trajectory of change that is observed in models of reorganization and those observed in studies of psychological therapy. The first is that the change trajectory observed in psychological therapy follows a *negatively decelerating* curve (67), which means that change is more likely to occur earlier in therapy than later. One early meta-analysis (68) suggested that given 75% of patients required 26 sessions for a successful outcome, this was a “rational” time limit. However, it is explicit that 75% of patients do not require this amount of input. Indeed, others have observed that therapy gains are greater in earlier sessions (69). Furthermore, this has led to the suggestion that change is nonlinear, dynamic and complex (70). However, the conclusion that change looks complex does not mean it is generated by a complex system. Indeed, the simulations reported by Powers (54) contain very few systems interacting; yet, each iteration of a simulation generates a different pattern of change, and takes different periods of time to achieve stability.

As already noted, gains in therapy are most often observed early in therapy (71). They also follow a discontinuous pattern, appearing suddenly and unexpectedly in many cases. This has been reported in naturalistic settings (72) and experimental studies (73). One approach to psychotherapy change is the dynamic system approach (74) that describes how client-therapist dyads produce new information that results in the “reorganizing of behavior”, which can occur suddenly. However, the algorithms underlying this model are arguably not straightforwardly linked to psychotherapy change, as the putative variables in the model are therapist interventions or working alliance, which are distal to the client experience of distress. The PCT account of therapeutic change considers chronic error, maintained by conflict, to be the crucial variable to simulate in modeling.

The PCT account of psychological distress is key for developing a parsimonious method of implementing computational models of psychotherapy change processes. The core trans-diagnostic process of conflict maintaining error across disorders has a clear implication: it means that a single approach to modeling can be used across the themes that people describe as problematic (e.g., isolation, evaluation of others, contamination, or maintaining weight). The account of psychological distress described above points to chronic error (loss of control) as crucial to understanding the underpinning symptoms. On this basis, all varieties of symptoms can be thought of as expressions of chronic error and, for this reason, simulations based on PCT principles generate a fundamental indicator of outcome—the magnitude of chronic error. If error is equivalent to psychotherapy outcome, simulations can be used to compare real-world therapeutic outcome with simulations based on PCT and this may be done across diagnostic categories.

As noted earlier there are some well-established findings in the psychotherapy literature of the trajectories of change, such as early gains, sudden gains, and the negatively decelerating curve. If error can be equated with outcome then it is possible to compare these patterns of outcome with the trajectory of change generated by models based on PCT principles. The model that provides a starting point for this investigation is the “trial-and-error” process described by Marken and Powers (66). The “trial-and-error” process is acknowledged to be fundamental to psychotherapy, as therapists are responsive to interventions that are ineffective by moving to new strategies (75). However, it is rarely acknowledged that clients are also exploring their mental life in therapy sessions, generating new perspectives and solutions; this is central to the PCT account of psychotherapy change. However, the implications of “trial-and-error” exploration for the trajectory of psychotherapy outcome have not yet been examined.

As already noted, the simplest starting point is to assume their symptom score at each session expresses the client’s current state of error. Each session is assumed to provide an opportunity to reorganize goals and generate novel solutions that reduce chronic error. The simulation of iterative episodes of reorganization has already been implemented in the PCT model described by Marken and Powers (66) and can be arranged to simulate changes in an outcome measure during successive sessions in psychological therapy. Like Marken and Powers (66), the model of therapy change can simulate successive iterations of reorganization with each iteration taken to reflect a “session” with severity of symptoms carried over from the last. The model can be set up so the output of the system changes to a greater extent when error is highest and less when error is lower. The output of the system would be analogous the activity people engage in when controlling a perception of, for example, a sense of connectedness to others. Thus, exploration is greatest when error is high. However, the exploration may be focused on a specific perspective on the problem, for example, thinking continually about how a social interaction might have gone differently so that humiliation did not occur. The forgoing account of the change process of therapy suggests that people can be helped to direct this exploration to higher-level perceptions.

**Figure 3:**
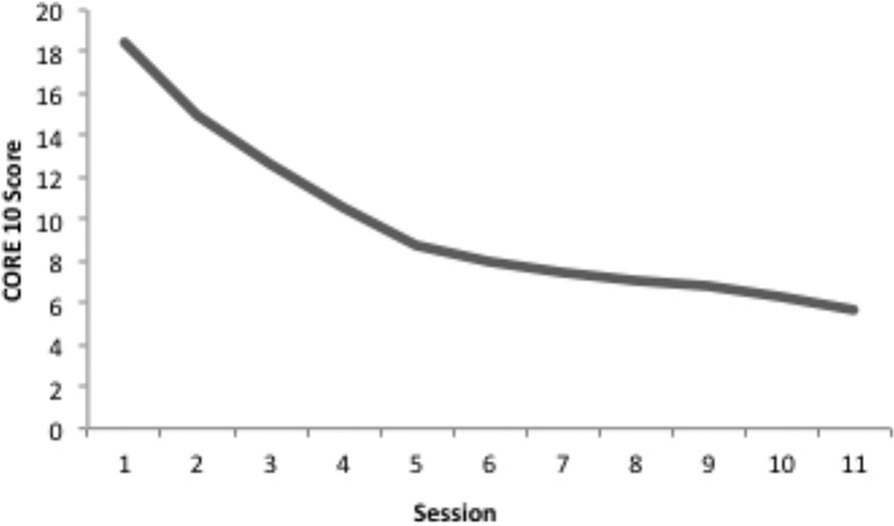
The trajectory of CORE 10 scores for a simulation based on the principle of reorganization. Original figure.

This verbal account of therapy can be cast as a computational model where exploration is conceptualized as a trial-and-error process constrained by the degree of error. On each “session”, the there is a reference value for a desired amount of symptoms (zero), and this is compared to current symptoms to generate an error signal. This error signal tunes the amount of random change in output (or behavior), greater error results in a higher chance of change in the system and vice versa. The output then has an effect on the environment that is either perceived as being either closer, or further, from the desire state of the perception and so another error signal is generated. In the model, error signal is taken to reflect the level of “symptoms” and this is standardized to a clinical dataset to observe the trajectory of change produced. **Figure 3** demonstrates a simulation of CORE 10 scores in N = 5,613 “cases” based on a starting point of the level of severity of symptoms taken from data reported by Stiles et al. (71). As can be seen in **Figure 3**, change is more likely early in the change process simulated by the model than later, generating the negatively decelerating curve described previously.

## Combined Computational Models to Show the Reorganization of a Higher-Level Control System Responsible for Conflict

While it is persuasive to see the qualitative parallels between the behavior of models of principles, such as conflict and reorganization, and the pattern of change in psychological distress, it does not directly test the specific mechanism thought to mediate change according to PCT. There are two steps in this advancement. First involves constructing a hierarchical model in conflict that regains control through reorganization of the higher-level system setting the two (or more) conflicting subgoals. Second involves constructing and optimizing these models for individual human participants and testing them against the real-world data as each participant experiences psychological change in therapy. Our research group has made significant progress with the first stage, and we are building the elements to carry out the second stage over the coming years.

In order to complete the first stage, we developed a bespoke software platform using C++ to allow the user to construct hierarchies of control systems that could be prone to conflict, and that could employ reorganization (76). The coding for this software is available from the first author, and second bespoke platform is also available from perceptualrobots.com.

Our first study tested whether allowing reorganization allowed two goals in conflict to regain control. It also tested the key prediction of a PCT model of psychological change—whether the restoration of control was more effective if the reorganization was directed at the higher-level goal setting the goals in conflict.

The control systems were constructed such that they would simulate the parallel processing that occurs in the nervous system, and they were refreshed every 16ms to allow dynamic updating to occur across all systems simultaneously. The experimenter optimized the parameters for each of the components of a single control system (e.g., gains and disturbances) so that this unit could control a single variable. Next, two higher level systems were now added to set the reference value for this system; the relative weighting (connection strengths) of these two systems in setting the lower level reference value was set randomly for 20 different agents. Next, a third level system was added to the top and it had two outputs—one setting the reference value for each of the two mid-level units. Again, these two connection strengths were set randomly for each of the 20 agents. In these simulations, reorganization worked by selecting a new direction and value of change in connection strength that was proportional to increase in cumulative error (loss of control) over a specified time window.

**Figure 4:**
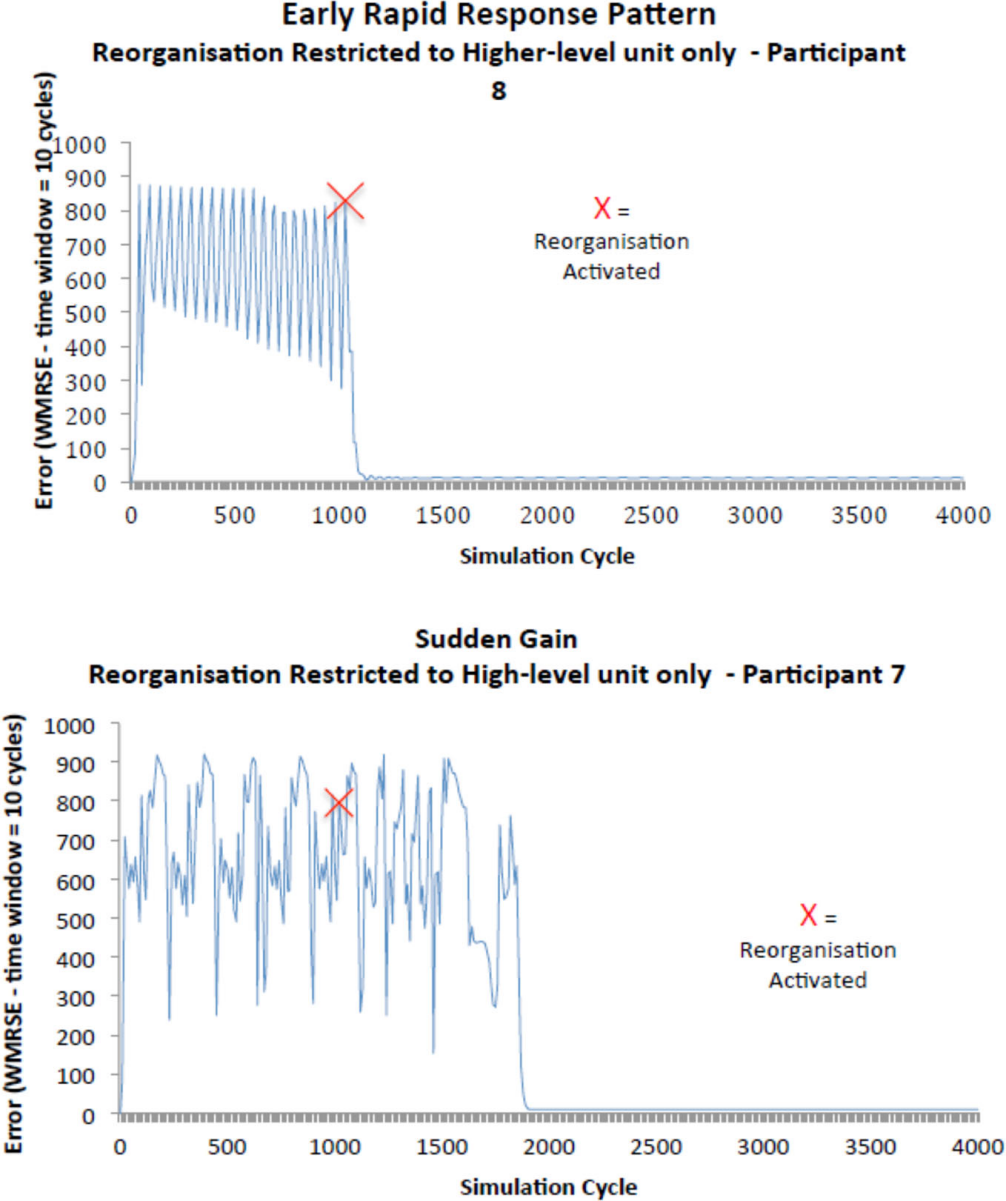
Graphs of change in overall error during the simulation in individual agents. The first pattern resembles the early response pattern found in patients, and the second pattern resembles sudden gains during therapy (77). WMRSE is the Window Mean Root Squared Error. To calculate WMRSE, the squared error terms across all control units are averaged over ten iteration cycles of the simulation, and then the square root of this figure is calculated.

The study tested the degree of restoration of control in three different conditions: no reorganization; reorganization of the mid-level systems; and reorganization of the higher level system. Akgonul (76) found, as predicted, that the agents who could reorganize regained more control, and that this effect was stronger when reorganization was limited to the higher-level system. This finding was replicated using the same platform in another study (77). The graphs of individual agents from this study also reveal the patterns of observed change in psychological therapy described earlier—the early response pattern, and sudden gains during therapy (see **Figure 4**). Thus, from a qualitative perspective, the PCT simulation shows similar patterns of psychological change to those that have been identified within individual patients.

## A Robust Computational Model of Psychological Change According to PCT

In order to prepare for the second stage and test a PCT model of psychological change against real-world data, a number of steps are required: (a) to identify an experience of psychological distress for which a clinically meaningful controlled variable can be easily measured, and for which a change would be expected after therapy; (b) to construct a simulation of the conflicted control systems governing this variable that can reorganize in the way specified by PCT; (c) to build this model and optimize it for individual participants; (d) to test the optimized models of individuals against the real-world data both before and after therapy.

**Figure 5:**
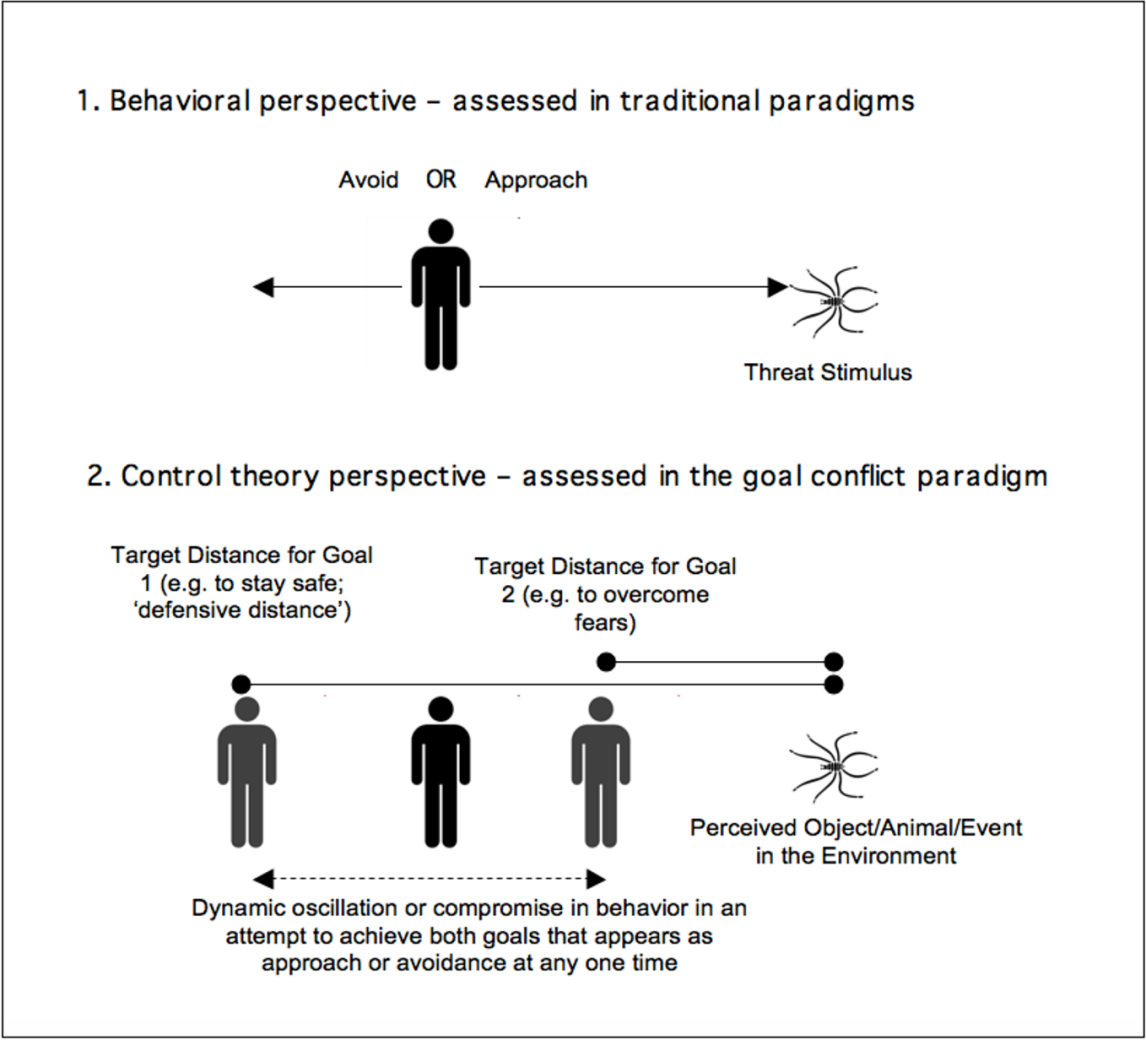
The behavioral perspective on avoidance contrasted with the control theory perspective (79).

Note. Traditional paradigms present a threat stimulus and measure whether avoidance or approach is triggered in the participant. A control theory perspective regards approach and avoidance as the observable consequences of an individual who has conflicting goals regarding their desired distance from an object in the environment (that may or may not be feared). Critically, when the defensive distance is greater than the distance required to fulfill another important goal (e.g., to overcome their fears; to spring clean their house; to return to work, etc.) the person maintains a compromise distance and any oscillation in behavior at each moment appears to the observer as either approaching or avoiding the object. Relatively strong fears in the context of weaker approach motivations would involve a compromise distance that is closer to the defensive distance.

We have selected spider phobia as the psychological problem to investigate. It is common and therefore easy to recruit, and the source of distress is relatively more circumscribed that other anxieties. Furthermore, it is commonly accepted that the distance that a person with spider phobia is willing to stand from a spider is an appropriate index of their difficulties that reduces with effective treatment (78). Yet, the control theory perspective on approach and avoidance differs from the behavioral perspective in explaining this process (see **Figure 5**). Specifically, “approach” and “avoidance” are not regarded as learned responses to the “stimulus” of a spider. Rather, according to PCT, the individual attempts to control a range of perceptual variables that are relevant to spiders, one of which is likely to be the desired distance. The same individual can have two discrepant desired distances from the spider. First, the defensive distance keeps the spider at a distance perceived to be sufficiently safe, and a second, closer distance, is set by higher level systems for goals such as “to recover from my phobia”, “to be strong”, or “to enjoy being in the garden”. Thus, what appear to be discrete, triggered behaviors to approach or avoid a stimulus are actually the oscillations between two conflicting perceptual set points—one close to, and one far away, from the feared entity—the spider in this example.

**Figure 6:**
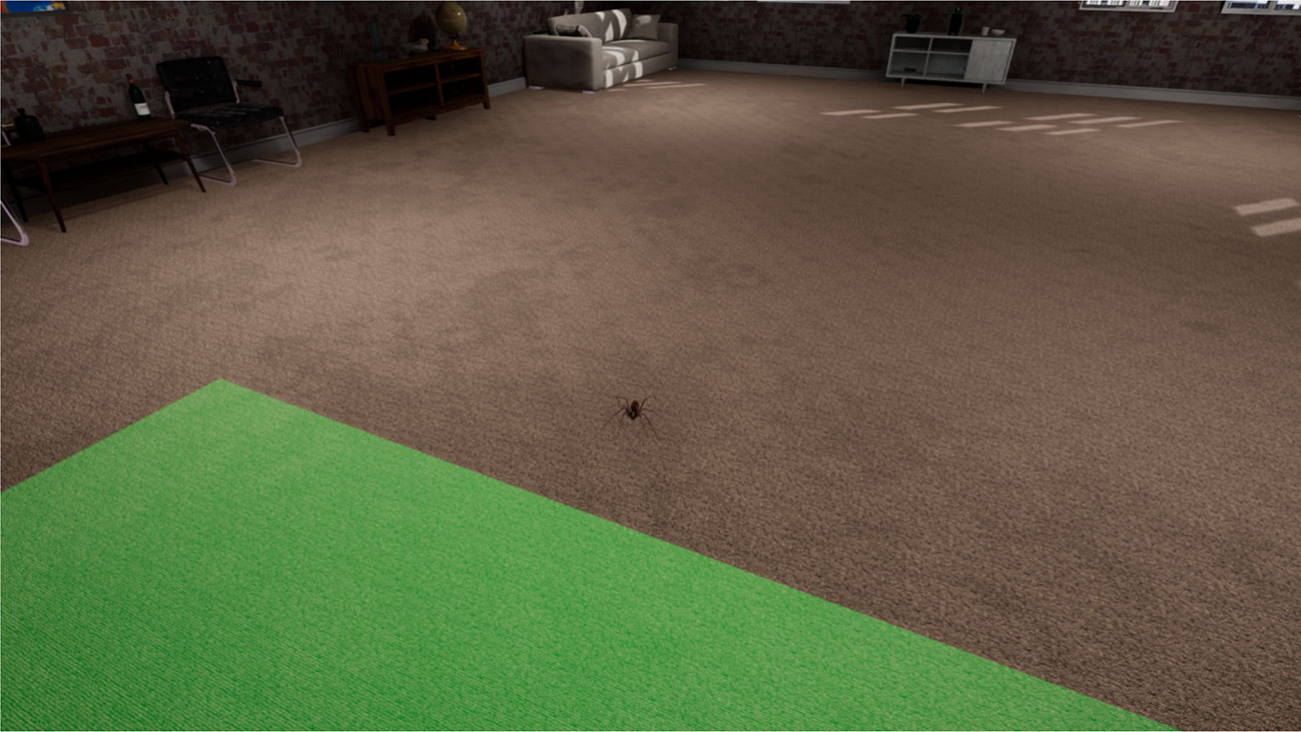
A static screenshot from the virtual reality environment within which each participant can control their distance from a naturalistically moving spider. Original image.

In order to assess the dynamic control of distance from a naturalistically moving spider in real time, we have developed a virtual reality paradigm in which the user can exert control over their distance from a spider in a room (see **Figure 6**). We have also developed a goal interview to allow each participant to report the importance of their conflicting goals with respect to their goals to be far away from the spider versus to be close to it (27). This interview also estimates the extent to which people with spider fears are aware of their goal conflict. There are also ways to attempt to assess goals and their conflicts using measures such a goal matrices (80) and repertory grids of personal constucts (81).

**Figure 7:**
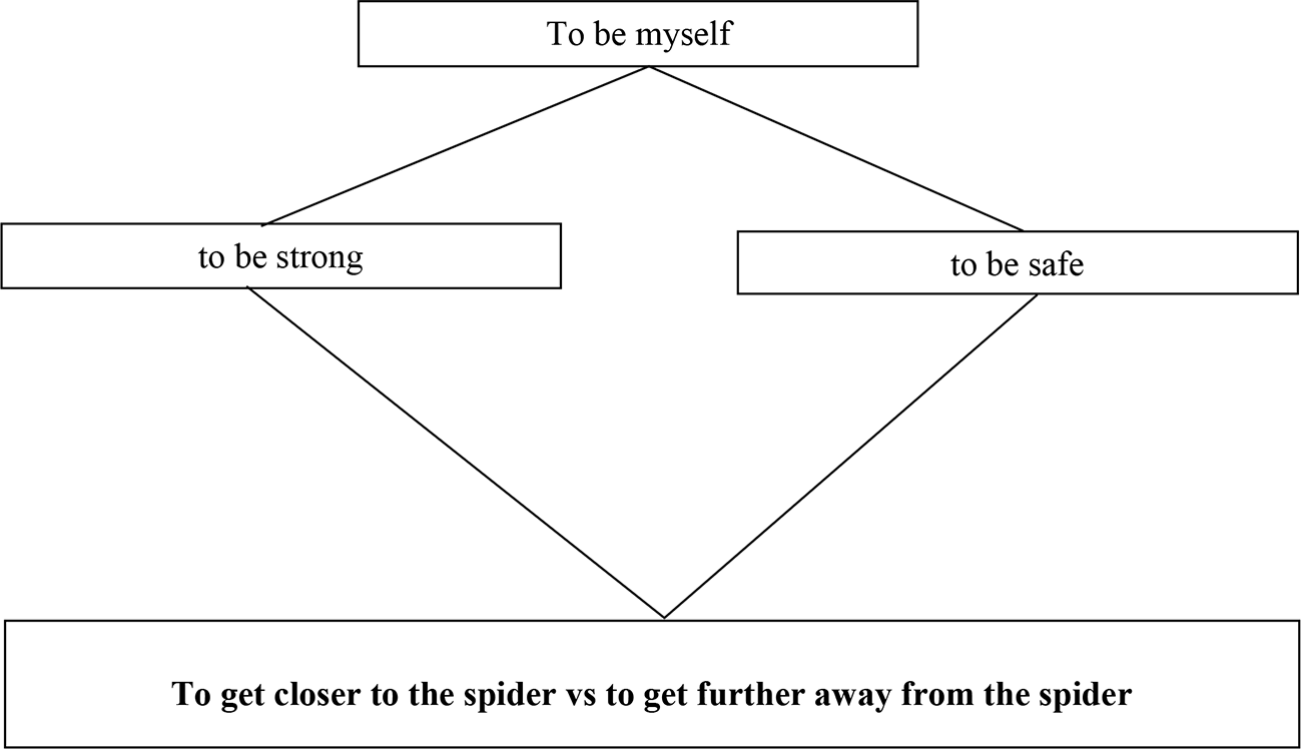
A simplified diagram of the three-level hierarchy involved in spider phobia. The fearful individual fluctuates in their distance from the spider depending on whether they are acting “to be strong” and get closer, or “to be safe” and get further away. However, when the same individual is provided with the opportunity to talk about their conflict and shift and sustain their awareness to the level setting these goals—”to be myself”—this permits trial-and-error reorganization of the higher-level system (e.g., sets different gains for each of the mid-level goals) such that the conflict is reduced and control increased.

We plan to construct a separate three-level hierarchical model for a large number of individuals with widely varying fears of spiders (see **Figure 7**). The initial gains for the control systems in conflict will be approximated as the importance ratings from the goal interview. Then each hierarchy will be optimized to each individual by training it on dynamic spider-distance data within the virtual environment over a fixed session length. In the first test of the validity of the model, we will measure its fit for each participant to the movements in a second VR session, thereby assessing the individual specificity of each model (33). Because goal conflict is likely to occur in some participants, the kind of exact match to behavior observed in tracking studies will not be possible for every individual. Nonetheless, there will be ranges of parameters for which behavior will be more predictable, for example where one goal has a much higher gain and therefore dominates the control of distance from the spider.

After establishing individual specificity, we aim to test the PCT model of psychological change. As in Akgonul (76) and Cooray (77), each hierarchy will be programmed to have the capacity to reorganize. Owing to the stochastic nature of reorganization, it will not be possible to assess the model fit against moment-by-moment behavior. Therefore, our aim is to show whether incorporating reorganization into the model can demonstrate the patterns of change that are shown within spider-fearful individuals who are exposed to the spider within VR; namely, the sudden reduction of distance from the spider after reorganizing the higher-level goals driving the conflicted systems. Indeed, our previous research has shown that people who report greater awareness of goal conflict extracted through an interview tend to approach closer to a spider after an exposure session (40). Therefore, we will optimize a reorganization parameter within the model of each participant to assess whether it can lead to the same pattern of approaching closer to the spider after the goal interview that we record in participants.

## Limitations, Extensions, and Future Developments

We have described our plans to test a simplified model of psychological change based on PCT. At present, it omits a number of features that are nonetheless known to be involved in psychological therapy and whose function is specified in PCT. These are: the biological (intrinsic) control systems within the individual; the physical environment within which action occurs; the therapist (for a diagrammatic PCT model of the therapy dyad, see 82); memories as perceptual goals (83); awareness (47); and communication through language (84). Therefore, there still remains a wide chasm between the details of a theory that can be described, and the extent to which all of these can be reproduced together within a faithful working model.

One novel solution to this issue is to use human actors to simulate individual clients before, during and after therapy in a manner similar to Stanislavski’s method of training actors [(85); Scholte, personal communication]. This method involves inferring a person’s multiple goals and their conflicts, acting according to them within a situation, and revising these goals iteratively to steadily improve the match to the script, or, in this case, the real-world behavior. This approach has advantages in terms of the potential complexity of goals that can be modeled, but it is clearly not specified computationally, nor can it provide robust tests of how the principles of a theory operate (e.g., reorganization).

This issue raises a further question around mechanism—not only is it important to test a functional model of the change process, but it is also necessary to test whether the human brain can implement this process, and if so, exactly how. There is likely to be a specific neuranatomic circuit involved in this process of resolving conflict: identification of conflict *via* the anterior cingulate, and two cortical structures—the ventromedial frontal cortex and orbital frontal cortex—that appear to be involved in evaluating the appropriate decision during goal conflicts [e.g., (86)]. However, a realistic neural model of this process that maps onto the PCT architecture we have described remains a future challenge.

The approach we have described has potential for areas of research into human behavior that extend beyond psychotherapy. For example, Bowlby’s attachment theory involves a conflict between exploration and safety-seeking, which can be specified as perceptual goals. It comes much closer to PCT because it is based on control systems theory, it involves interpersonal dynamics, and its internal working models are essentially hierarchical and conflicting control systems (87). The use of robots to model the dynamics of child-caregiver interactions in earlier studies [e.g., (88, 89)] could be extended to the interactions between therapist and client during exposure, for example, and could be optimized to test against behavioral data from humans. The fields of social and personality psychology have also touched upon dynamic modeling, but could be expanded upon (90). It is also feasible that research on robotics could eventually utilize and test PCT models of psychological change, involving memory and learning, following promising work on simulating navigation and locomotion (91).

## Summary

We have described a novel and sophisticated methodology to test the mechanism of psychological change within psychological therapies. This approach requires the construction of computational models that attempt to directly emulate the psychological mechanisms occurring within each individual client. We summarized the evidence to date, which is consistent with a PCT account of psychological distress as chronic, unresolved goal conflict held outside awareness. However, a robust evaluation of this model requires a long-term research program that is currently in progress. It is our view that researchers need to become acquainted with this methodology and to start to develop the skills and resources to use it, because it provides a feasible way to properly “understand the engine” of psychological change, and how best to engage and harness it within clinical practice.

## Author Contributions

WM conceived of the review and organized the structure and main argument, and WM and VH produced the content.

## Conflict of Interest

The authors declare that the research was conducted in the absence of any commercial or financial relationships that could be construed as a potential conflict of interest.

The handling editor is currently co-organizing a Research Topic with one of the authors, WM, and confirms the absence of any other collaboration.
